# TWEAK Appears as a Modulator of Endometrial IL-18 Related Cytotoxic Activity of Uterine Natural Killers

**DOI:** 10.1371/journal.pone.0014497

**Published:** 2011-01-07

**Authors:** Marie Petitbarat, Mona Rahmati, Valérie Sérazin, Sylvie Dubanchet, Corinne Morvan, Robert Wainer, Philippe de Mazancourt, Gérard Chaouat, Jean-Michel Foidart, Carine Munaut, Nathalie Lédée

**Affiliations:** 1 INSERM, U782, Implantation et Dialogue Materno-Fœtal, University Paris-Sud, UMR-S0782, Hôpital Antoine Béclère, Clamart, France; 2 Department of Reproductive Medicine and Molecular Biology, EA 2493, CHI Poissy/St Germain en Laye, University of Versailles Saint Quentin en Yvelines, Poissy, France; 3 Laboratory of Tumor and Development Biology, CHU Sart Tilman, University of Liège, Liège, Belgium; Massachusetts General Hospital and Harvard Medical School, United States of America

## Abstract

**Background:**

TWEAK (Tumor necrosis factor like WEAK inducer of apoptosis) is highly expressed by different immune cells and triggers multiple cellular responses, including control of angiogenesis. Our objective was to investigate its role in the human endometrium during the implantation window, using an ex-vivo endometrial microhistoculture model. Indeed, previous results suggested that basic TWEAK expression influences the IL-18 related uNK recruitment and local cytotoxicity.

**Methodology/Principal Findings:**

Endometrial biopsies were performed 7 to 9 days after the ovulation surge of women in monitored natural cycles. Biopsies were cut in micro-pieces and cultured on collagen sponge with appropriate medium. Morphology, functionality and cell death were analysed at different time of the culture. We used this ex vivo model to study mRNA expressions of NKp46 (a uNK cytotoxic receptor) and TGF-beta1 (protein which regulates uNK cytokine production) after adjunction of excess of recombinant IL-18 and either recombinant TWEAK or its antibody. NKp46 protein expression was also detailed by immunohistochemistry in selected patients with high basic mRNA level of IL-18 and either low or high mRNA level of TWEAK. The NKp46 immunostaining was stronger in patients with an IL-18 over-expression and a low TWEAK expression, when compared with patients with both IL-18 and TWEAK high expressions. We did not observe any difference for TWEAK expression when recombinant protein IL-18 or its antibody was added, or conversely, for IL-18 expression when TWEAK or its antibody was added in the culture medium. In a pro-inflammatory environment (obtained by an excess of IL-18), inhibition of TWEAK was able to increase significantly NKp46 and TGF-beta1 mRNA expressions.

**Conclusions/Significance:**

TWEAK doesn't act on IL-18 expression but seems to control IL-18 related cytotoxicity on uNK cells when IL-18 is over-expressed. Thus, TWEAK appears as a crucial physiological modulator to prevent endometrial uNK cytotoxicity in human.

## Introduction

The endometrium is remodelled throughout the menstrual cycle and exhibits only a short period of receptivity, known as the ‘implantation window’, which is crucial both for implantation and gestation and still remains poorly explored in routine reproductive medicine. Endometrium becomes receptive to blastocyst implantation 6 to 8 days after ovulation and remains receptive for 4 days (cycle days 20–24) [1]. Such differentiation of the maternal compartment, under hormonal control, is essential to allow stromal cells to acquire the unique ability to regulate trophoblast invasion, to resist inflammatory and oxidative insults, and to dampen local maternal immune response. In humans, decidualization of the stromal compartment occurs in the mid-luteal phase of the menstrual cycle, independently of pregnancy, in contrast to most of animal models. This raises the possibility that biochemical analysis of timed endometrial biopsy samples taken in a nonconception cycle could be informative of subsequent pregnancy outcome [2].

It has been proposed that uterine natural killer cells (uNK) could exert, directly or not, a positive or negative control of the early steps of implantation [3], [4]. These cells can secrete and control an array of cytokines which are important in angiogenesis, placental development and in pregnancy establishment. A dysregulation of these cytokines could be in part responsible of embryo implantation failures. In previous studies, we reported that IL-15 and IL-18 expressions were significantly different in patients who failed to implant when compared with fertile control individuals [5]. We also observed that IL-15 and IL-18 expressions were correlated with the local uNK (CD56+) recruitment and subendometrial angiogenesis as reflected by the vascular flow index quantified by 3-D ultrasound with angiography [6].

TWEAK (Tumor necrosis factor like WEAK inducer of apoptosis) is a transmembrane protein, which can be cleaved to function as a soluble cytokine [7]. It's highly expressed by immune cells type (monocytes, dendritic cells, natural killers cells…) and many tissues [8].

First described as a weak apoptosis inducer, TWEAK triggers multiple cellular responses [9] ranging from proliferation to cell death, including control of angiogenesis [10], [11], [12]. TWEAK has also been described as a partner to TNF (Tumor Necrosis Factor) playing a « Yin and Yang » function in immunity [13].

We recently reported that TWEAK and IL-18 mRNA expression were correlated in patients with implantation failures [14]. Therefore we investigated whether TWEAK regulates IL-18 expression or at the opposite if IL-18 acts on TWEAK expression. TWEAK mRNA and protein expression does not show variations through the menstrual cycle.

However its basal level of expression influence the IL-18 related uNK recruitment and local cytotoxicity. Indeed, IL-18 is a bivalent cytokine which can promote local angiogenesis and immunotrophism at appropriate dose but conversely promote a cytotoxic and thus deleterious commitment of uNK cells if present in excess [5]. Such documentation appears to be essential to define the state of uterine receptivity.

Endometrial dispersed cells cannot be used to document the *in vivo* environment since this technique does not maintain the interactions between epithelial cells, stromal cells, endothelials cells and immune cells (uterine natural killer cells, T regulatory cells and dendritic cells). The development of a human *ex vivo* model in which the complexicity of endometrium is preserved as well as its functionality is an absolute requirement. In order to study stromal-epithelial interactions in the human endometrium, many authors elaborated culture conditions recombining separated stromal and epithelial cells in different extracellular matrices [15], [16], [17], [18]. In such models, epithelial cells have been cultivated as monolayers on top of matrigel and stromal cells beneath them on plastic or embedded in collagen or Matrigel. Other methods were described using polycarbonate or nitrocellulose culture of tissue insert [19], [20] and demonstrated human epithelial and stromal cells were viable during 1 to 5 days. In all these models, cells viability was demonstrated but neither of them appears fully appropriate to study the expression and production of cytokines or growth factors in endometrial cells as well as to mimick the complexicity of their interactions with immune and endothelials cells.

We thus decided to develop a model of endometrial microhistoculture, adapted from the one elaborated by Menu *et al* [21] for the placenta, in which the complex interactions between epithelial, stromal and immune cells were preserved and the viability and responsiveness of the cells are verified. Using this model, we investigated whether TWEAK influences the activities of uNK cells by a modification of IL-18 expression or function.

## Results

### Validation of microhistoculture model

**Figure 1:**
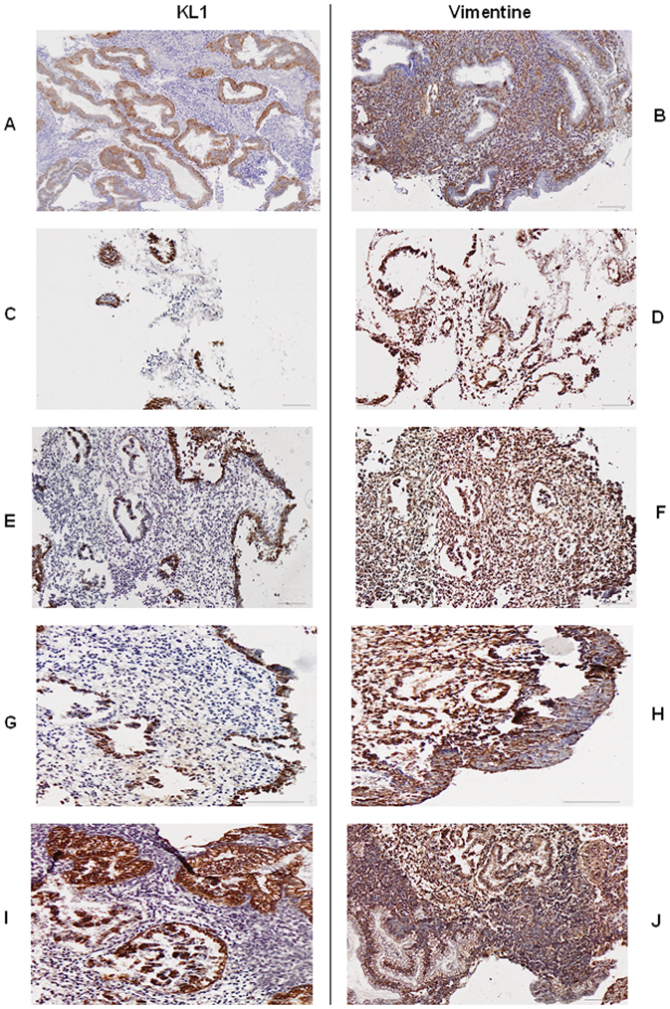
Endometrium cells morphology providing from luteal phase. Revealed by immunohistochemistry with cytokeratin (KL1) and vimentin antibodies (x 6,6). A–B: before culture, C–D: after 2 days of culture without hormones and without sponge, E–F: after 2 days of culture without hormones and with sponge, G–H: after 2 days of culture with hormones (50 nmol/l estradiol +50 nmol/l progesterone) and without sponge, I–J: after 2 days of culture with hormones (50 nmol estradiol +50 nmol progesterone) and with sponge.

**Cells morphology:** The main problem we encountered with endometrium *in vitro* histoculture was to maintain differentiated cells and avoid they become fibroblastic. We analysed the endometrium structure as well as the morphology of cells by immunohistochemistry using two different antibodies: vimentin (endothelial cells staining) and KL1 cytokeratin (epithelial cells staining). We compared the morphology at different days of culture with or without estradiol and progesterone addition (50 nmol/l) and with or without use of collagen sponge (Figure 1).

In culture without sponge use, stromal cells are flat and elongated (Figure 1C and D) compared to those cultured with sponge which remained rounded (Figure 1E and F). But in both case epithelial cells are dispersed. Cells cultured with estradiol and progesterone (Figure 1G–J) displayed a well preserved morphology, where epithelial cells maintain their polarisation. We realised the same experience using endometrium biopsies from proliferative phase and observed the same results (data not shown). Thus, to conserve their morphology endometrial cells must be cultured on collagen sponge with hormones addition in medium.

**Figure 2:**
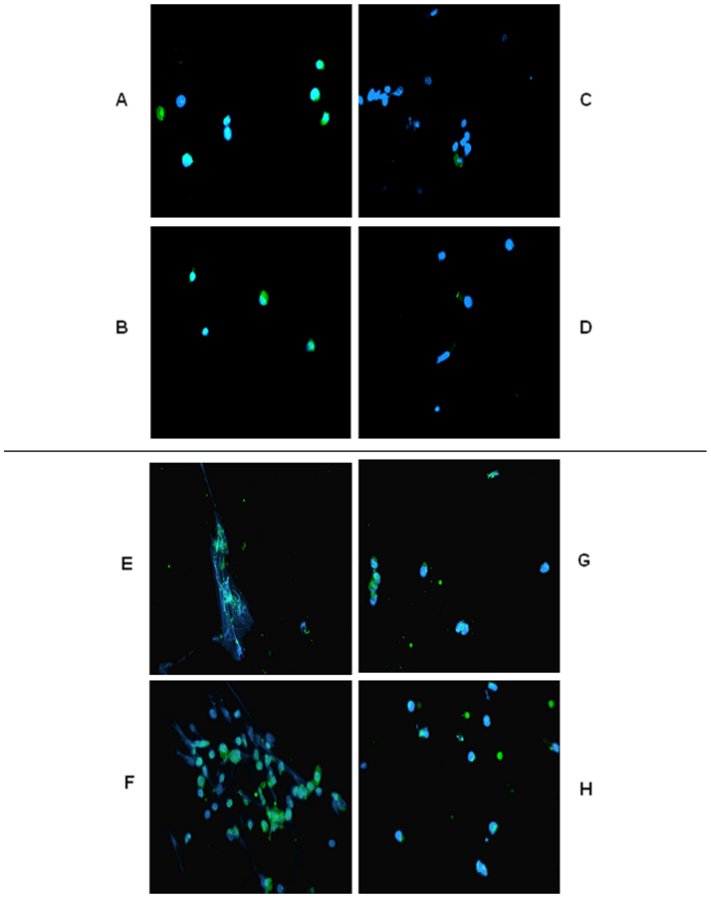
Endometrium cells death revealed by FITC-Annexin V antibody. A–D: after 2 days of culture, E–H: after 5 days of culture. A,E: without sponge and without hormones, B,F: without sponge and with hormones (50 nmol/l estradiol +50 nmol/l progesterone), C,G: with sponge and without hormones D,H: with sponge and with hormones (50 nmol/l estradiol +50 nmol/l progesterone).

**Cells death:** Cells viability was evaluated by immunohistochemistry using Annexin V Fluos, an antibody coupled to FITC (Figure 2). The Annexin V assay provides effective method to detect apoptosis at a very early stage. After the induction of apoptosis phosphatidylserine is translocated from the inner (cytoplasmic) leaflet of the membrane to the outer (cell surface) leaflet, phosphatidylserine on the outer leaflet is available to bind labeled Annexin V, providing the basis for a simple staining assay.

After two days of culture we only observed Annexin V staining on cells growing without collagen sponge matrix (Figure 2A and B) compared to those culture with Gelfoam sponge (Figure 2C and D). After five days, all cells cultured with or without sponge are labeled (Figure 2E–H). Besides, after five days of culture without sponge cells appeared elongated and fibroblastic whereas cells growing with collagen sponge matrix are round.

We didn't observe any difference on cells viability when we added estradiol and progesterone in culture medium.

**Cells functionality:** We assay in supernatants, by Luminex Technology, after 2 and 3 days of culture 8 cytokines and growth factors: interleukine 1β (IL-1b), interleukine 1 receptorα (IL-1Ra), granulocyte colony stimulating factor (G-CSF), interferon inducible protein-10 kilodaltons (IP-10), monocyte chemoattractant protein 1 (MCP-1), macrophage inflammatory protein 1β (MIP1b), platelet-derived growth factor β (PDGFb) and regulated on activation of normal T cell expressed and secreted (RANTES). These molecules were chosen as « markers » of endometrial activity cells because their expression were different between flushing with endometrial cells and flushing with no cells (data not shown). We previously observed that addition of estradiol and progesterone are suitable to maintain a well preserve structure of epithelial cells. Our objective was to validate a model to study expression or production of bio-molecules. So it was important to determine whether estradiol and progesterone supplementation influence cytokines or growth factors production.

**Figure 3:**
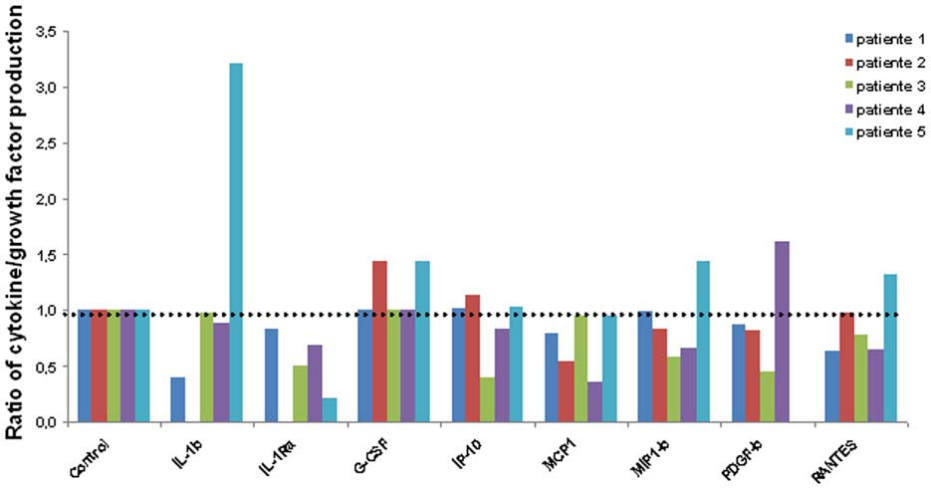
Cytokine and growth factors measures in five different patients. The culture medium was supplemented with 50 nmol/l of estradiol and progesterone. Proteins levels were measured on days 2 and 3. The graph represents for each protein the ratio of day 3 measure on day 2 measure, reported to the control condition. The control condition had no hormone addition. IL-1b and IL-Ra for patient 2 and PDGF for patient 5 were not detectable.

We realised eight different conditions using 3 estradiol or progesterone concentrations (50 nmol/l, 100 nmol/l, 200 nmol/l) and a control condition without hormone. Cytokines and growth factors production was assayed after 2 and 3 days of culture. Because the cellular mass from endometrium sampling could not be standardised, cytokine/growth factor production by day could not be compared between individuals. So we chose to express our results by the ratio day 3/day 2 cytokines production. This ratio was measured by patients for each cytokine and each hormonal concentration compared to control (without hormones). No significant variation of cytokines and growth factors production was observed according to distinct concentrations of estradiol or progesterone or their comparison to control condition. Figure 3 represents the cytokine/growth factor production for minimal concentration of estradiol and progesterone (50 nmol/l) for each patient.

### Role and regulation of TWEAK in endometrial cells

A minimal concentration (50 nmol/l) of estradiol and progesterone was added in each culture condition for all the experiments.

#### Role of TWEAK on the uNK cytotoxic receptor NKp46

We compared the presence of the uNK cytotoxic receptor, NKp46, in patients with an IL-18 over-expression (if compared to the one observed in fertile patients) and either a low or high mRNA TWEAK expression.

**Figure 4:**
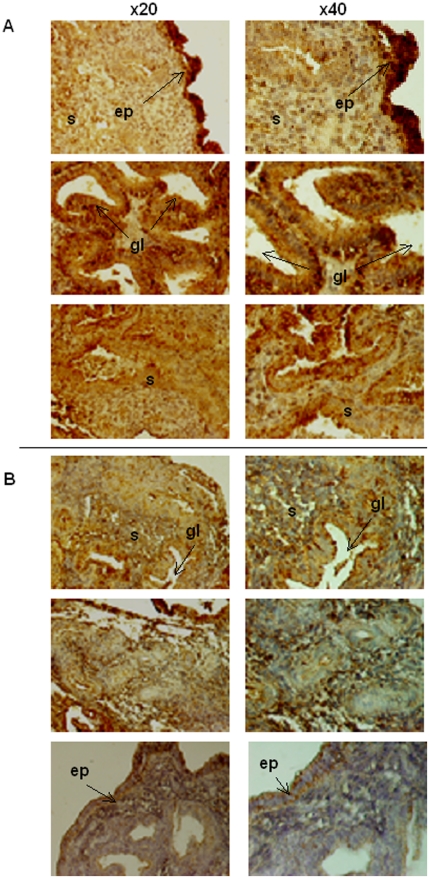
NKp46 immunostaining. Patients with an overexpression of IL-18 and with low (A) or high level of TWEAK (B). Magnification is 20X (first column) and 40X (second column). ep: epithelium, s: stroma, gl: glands, sa: spiral arteries.

The NKp46 protein was mainly detectable in the luminal and glandular epithelium and within the stroma around the spiral arteries. The NKp46 immunostaining was stronger in patients with IL-18 over-expression and simultaneously a low TWEAK expression (Figure 4A) if compared with patients with IL-18 and TWEAK over-expression (Figure 4B). No variation in staining localisation was observed. Presence of TWEAK seems to control the potential cytotoxic role of IL-18 on uNK cells, in case of over-expression.

#### Does TWEAK regulate IL-18 or the opposite?

We compared the expression of TWEAK and IL-18 under different stimulation to determine if TWEAK regulate IL-18 expression or be regulated by this cytokine.

**Figure 5:**
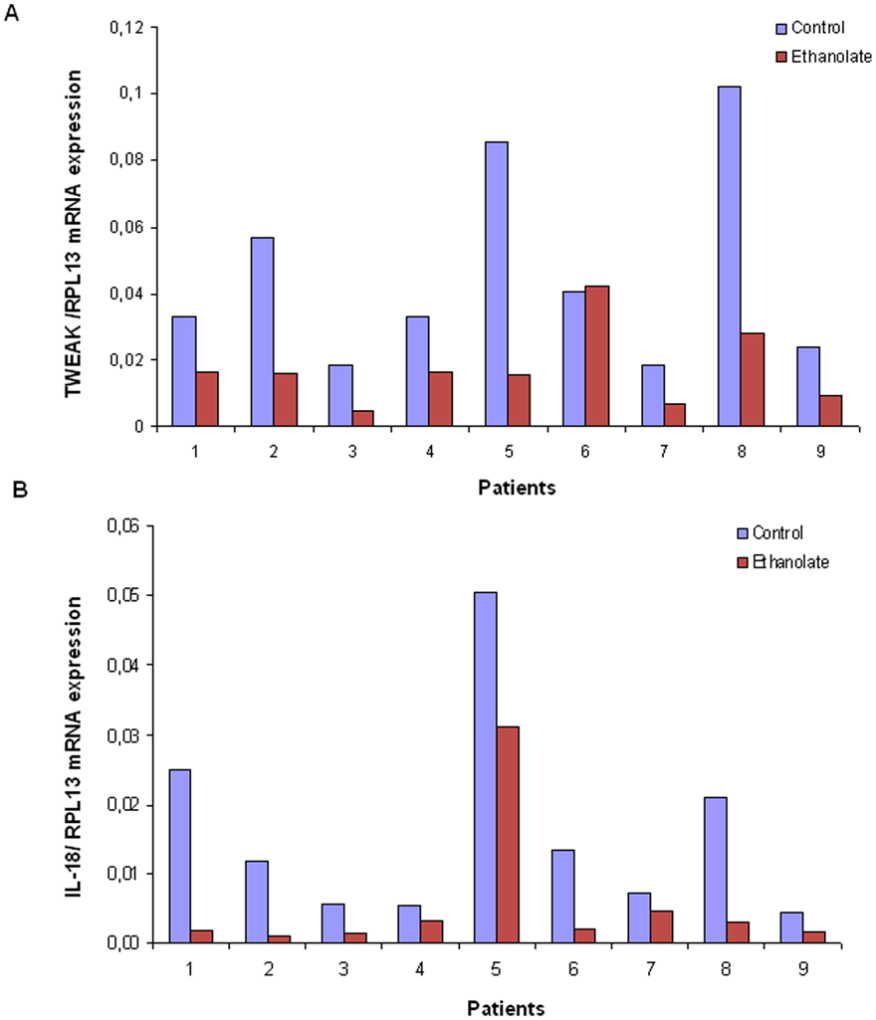
TWEAK (A) and IL-18 (B) mRNA expression. From endometrial microhistoculture in 9 patients. The control medium was only supplemented with 50 nmol/l of hormones (estradiol and progesterone). Ethanolate was added to the medium as an inhibitor of Map Kinase pathway. Estradiol and progesterone were added in all media.

Stimulation of recombinant protein IL-18 or its antibody in the culture medium did not influence subsequent TWEAK expression if compared to the control condition. Only addition of ethanolate as known inhibitor of the Map Kinase pathway induced a significant TWEAK mRNA decrease (*p* = 0.01) (Figure 5A). Likewise, there was no variation of IL-18 expression after 24 h of stimulation with recombinant TWEAK or its antibody in the culture supernatant. As for TWEAK, only addition of ethanolate produced an important IL-18 mRNA decrease (*p* = 0.07) (Figure 5B).

TWEAK expression is not regulated by IL-18 and IL-18 has no effect on TWEAK mRNA. These results highlighted TWEAK and IL-18 expression are independent one from the other in human endometrium but are both regulated via a Map Kinase pathway.

#### TWEAK and uNK cells

In order to verify whether TWEAK could counterbalance IL-18 cytotoxic effect on uNK cells, we quantified, in different culture conditions, the expression of two proteins: NKp46, which mediates uNK cells cytotoxic effects and TGF-β1 (Transforming Growth Factor–β) which promote the angiogenic effect of uNK cells.

**Figure 6:**
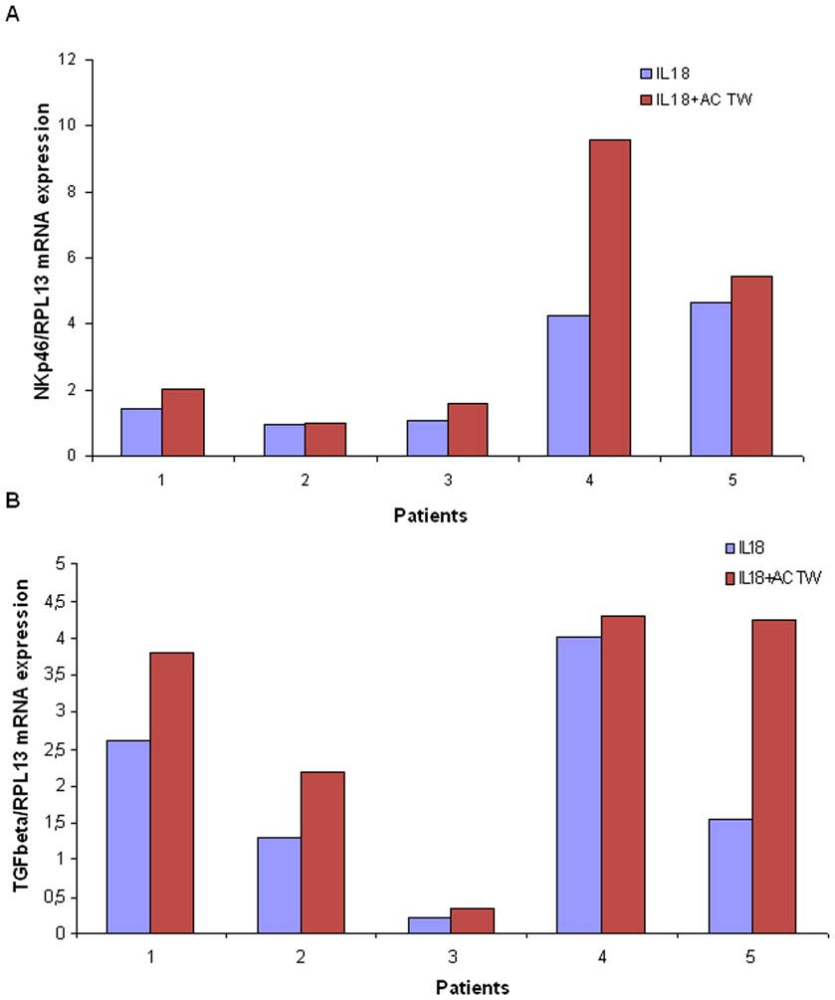
NKp46 (A) and TGF-β (B) expression. From endometrial microhistoculture in 5 different patients. Recombinant human IL-18 (IL-18) alone or with anti-human TWEAK (IL-18 + AC TW) were added to the medium. Estradiol and progesterone were added in all media.

We added in culture media an excess of IL-18 recombinant protein to create an endometrial pro-inflammatory environment in all culture conditions. Excess of recombinant TWEAK protein or its antibody was introduced in the medium too, in the aim to modify IL-18 effects. Addition of TWEAK in the culture medium had no effect on NKp46 or on TGF-β1 expression when compared to the IL-18 basal over-expressed condition. However, in TWEAK antibody supplemented culture medium, we observed a significant increase of both NKp46 (Figure 6A) and TGF-β1expression (Figure 6B). Only an imbalance between IL-18 and TWEAK production has an impact on the two protein expressions.

## Discussion

We demonstrated, using a collagen sponge gel support with hormonal substitution, that the endometrial cells are viable during four days, do not dedifferentiated and are able to produce endometrial “markers”. Indeed, the cells conserved their morphology and functionality even with a minimal hormone concentration. Our model was very simple to use and we maintained interaction between the different cellular types (epithelial, stromal, endothelial and immunological cells). It seems to be a good system to study the production or expression of cytokines and growth factors during the window implantation in women. With this model we show that IL-18 and TWEAK expression in endometrium are independent one from the other but share the same way of regulation, the Map Kinase pathway. However, as previously suggested, we confirmed that TWEAK acts on the uNK cells cytotoxicity, induced by IL-18 over-expression, by modifying one of the uNK cells cytotoxic receptor expression, NKp46.

The comprehensive functions of uNK cells still remain unclear. The two possibilities that are currently under investigation are that uNK cells influence maternal mucosal and arterial function [22] and/or placental trophoblast invasion [23]. This assumption is further supported by the high levels of angiogenic growth factor (Vascular Endothelial Growth Factor C, Placental Growth Factor, Angiopoietin-1 and 2, Transforming Growth Factor -β) secreted by uNK cells from both non-pregnant endometrium and early decidua [24].

In human, Laird *et al* observed an increased density of CD56^bright^ (uNK cells) in mid luteal phase endometrium in women suffering from recurrent implantation failures compared with fertile controls [25] but did not evaluate either their cytotoxic potential or cytokines content. As we previously reported that high IL-18/TWEAK endometrial mRNA expression correlated with a significant increase of uNK cells recruitment, we investigated the role of these two cytokines on uNK cells cytotoxicity. Manaster *et al*, reported that both proliferative and secretory phase uNK cells express NKp46 and NKG2D, but do not express NKp44 and NKp30, at the opposite of NK cells from decidua which express high levels of NKp30, NKp44 and NKG2D [26]. We then decided to choose NKp46 as target to evaluate cytotoxicity of uNK cells in endometrium. Our immunohistochemistry and PCR results show that TWEAK doesn't act on IL-18 expression, but seems to control IL-18 related cytotoxicity on uNK cells when IL-18 is over-expressed. Thus, TWEAK appears as a modulator to avoid endometrial uNK cytotoxicity induced by IL-18 over-expression.

Besides we reported that TWEAK also influence TGF-β1 cytokine production, The biology of the TGF-β family is complex and the various roles attributed to TGF-β in the uterus are still unclear. Our results revealed that when IL-18 is over-expressed and TWEAK is inhibited by a specific neutralising antibody, an increase of TGF-β1 is observed. We postulated that the observed TGF-β1 increase could reflect a retro-control loop; as TWEAK could not protect anymore endometrial cells from inflammation, others proteins with anti-inflammatory effect will be produced, like TGF-β. Eriksson *et al* recently reports that endometrial production of TGF-β regulate uNK cell proliferation but also cytokine production and in particular the IFN-γ production [27]. The combined endometrial IL-18 over-expression with a TWEAK inhibition increase cytotoxic receptors of uNK cells which produce massively pro-inflammatory cytokines like IFNγ. Local TGF-β are produced to counterbalance the cytotoxic activities.

In summary, the present study presents IL-18 and TWEAK as two independent proteins possibly acting in synergy to maintain the angiogenic/cytotoxic balance related to uNK cells. Such data should be controlled on larger number of patients. Taken together, these results strengthen the idea that IL-18 on TWEAK mRNA expression should be evaluated as functional biomarkers of an adequate/inadequate uterine receptivity in order to detail the mechanisms of failures.

## Material and Methods

### Patients

All patients provided informed consent and this investigation was approved by our Institutional Review Board (CCPPRB). 30 women were enrolled in this protocol, 21 were Caucasian, 6 were from North of Africa and 3 from Africa. All subjects were involved in the Assisted Reproductive Technology program for investigations related to their infertility: 13 women experienced unexplained recurrent spontaneous abortion and 17 repeated and unexplained implantation failure following *in vitro* fertilization (IVF). They have no other remarkable pathological profile. 6 patients in this group had given birth to healthy infants. The inclusion criteria included a normal hormonal reserve (FSH<10 mIU/ml) and patients were less than 38 years old (with a mean of 34,54).

8 patients were included to document NKp46 immunostaining: all patients had a high IL-18 mRNA expression and either a low (4 patients) or a high (4 patients) TWEAK expression. 22 other patients were included for the microhistoculture experiment. Individual analysis of each endometrial immune environment was detailed as previously described [28] and showed either a normal local uNK recruitment and activity or a cytokine–related uNK depletion. None had a pro-inflammatory profile before the microhistoculture in this group.

#### Endometrium Biopsy

Prior the endometrial biopsy, a systematic endometrial ultrasonic examination was performed to record the endometrial volume, exclude any intra-uterine abnormality of the uterine cavity and document the overall uterine vascularisation. As previously described [29], , a single operator (N.L.) employed an automated 5–9 MHz transvaginal three-dimensional transducer (Voluson 730 expert, GE Medical System, France) to acquire power Doppler information from the uterus using identical settings and technique in all cases as follows: tissue harmonic imaging, mild; pulse repetition frequency, 0.6 khz; wall motion filter, low; gain, −7.8; high quality, rise 0.2. The volumetric and vascular measurements were undertaken using the Virtual Organ Computer-aided AnaLysis (VOCAL™, GE Kretz) imaging 4-D VIEW program with the 15° rotation step. This allows the user to define the volume of interest manually and applies a shell of variable thickness that parallels the originally defined surface contour, thus allowing delineation of the myometrial-endometrial border. Intensity and quantity of the power signal was defined in the sub-endometrial volume (endometrial volume +5 mm from the border). Endometrial biopsies were performed (without anesthesia) during a monitored natural cycle 7 to 9 days after the ovulation surge (LH) identified by serial blood tests. Biopsies were performed with a standard Cornier pipelle (CCD Laboratories, Paris, France). This method collects material from the superficial layer and not the basal one, minimizing variations due to sampling from different depths. One portion of the sample was embedded in paraffin for immunostaining, another one was immediately placed in a tube with RPMI 1640 Glutamax medium at 4°C for endometrium histoculture. The last portion transferred into RNA Stabilization Solution (RNA Later, QIAGEN, Courtaboueuf, France) was used to quantify the basal level of TWEAK and IL-18 in fresh endometrium by real time PCR.

### Endometrial Micro-histoculture

5 to 9 patients were included in each experiment and each patient was her own control. The experiments were performed in triplicate. During the 2–3 hours following collection, endometrium biopsies were washed with RPMI 1640 medium. Collagen sponge gels (1.5 cm^2^, Gelfoam®, Pharmacia Upjohn, Kalamazoo, MI, USA) were placed into the wells of 12-well plates containing 1,5 ml per well of RPMI 1640 Glutamax medium supplemented with 15% heat-inactivated fetal calf serum (FBS), 1% penicillin–streptomycin, 1% non-essential amino acids, 1% sodium pyruvate (Gibco BRL, Life Technologies, France). Endometrium were cut into 1–2 mm blocks and placed on top of the collagen sponge gels at the interface between the medium and the air (3 blocks per collagen sponge and per well).

To validate the micro-histoculture model, four different conditions were realised with or without collagen sponge gel and with or without 50 nmol/l of β-estradiol and progesterone (Sigma, France). Estradiol and progesterone were added to the medium every day. Micro-histocultures were maintained at 37°C (5% CO_2_). For the experiments, we used the collagen sponge gel with 50 nmol/l of β-estradiol and progesterone. Antibodies, recombinant protein or inhibitors were added to the medium after 24 hours of culture. Biopsies pieces were collected after 24 hours of incubation with the recombinant proteins, antibodies or inhibitors and transferred into RNA Stabilization Solution (RNA Later, QIAGEN, Courtaboueuf, France) to be stored at −80°C. The RNA extraction was performed several days later. Recombinant human TWEAK (75 ng/ml), anti-human TWEAK antibody (400 ng/ml), recombinant human IL-18 (100 ng/ml) and anti-human IL-18 antibody (900 ng/ml) were from R&D sytems (Lille, France). Ethanolate (5 µmol/l), an inhibitor of MAP Kinase pathway was purchased from Sigma (St Quentin-Fallavier, France).

### Evaluation of cell death

The other part of micro-explants was placed into a well of 24 well plates with 1 ml of collagenase IV (1 mg/ml, Laboratoires Eurobio, Courtaboeuf, France) during 2 hours at 37°C. The dissociated cells were washed with PBS and incubated in a buffer containing 500 µl Hepes and 10 µl Annexin V-Fluos (Roche Diagnostics, Meylan, France) during 10 min at 37°C in darkness.

Cells were rinsed with Hepes and observed under a fluorescence microscope. Dapi (4′,6-diamidine-2-phenyl indole) was used to stain nuclear cells (CML, Nemours, France).

### Luminex

This experiment was realised using endometrial samples from 5 different patients. The culture medium was collected and fresh medium was added on days 2 and 3. Medium was frozen at -20°C until assay by the Luminex Technology (Bio-Rad®). This technology was used to simultaneously read concentrations of IL-1β, IL-1RA, G-CSF, PDGF, IP-10, MCP-1, MIP-1β and RANTES.The assay was performed according to the manufacturer's instructions.

### Total RNA extraction and reverse transcription

Endometrial fresh biopsy and micro-explants were disrupted in the lysis buffer of a RNeasy kit (QIAGEN, Courtabeuf, France) using an Ultra Turrax T15 (IKA-WERKE). Total RNA was extracted using the RNeasy kit according to the manufacturer's instructions. Additional DNase digestion was performed during the extraction process (RNase-free DNase set, QIAGEN, Courtabeuf, France).RNA was stored at −80°C until use. Total RNA (1 µg) was reversed transcripted into cDNA using random primers and Superscript III (Invitrogen, Carlsbad, CA) according to the manufacturer's instructions. Controls without reverse transcriptase were systematically performed to detect genomic DNA contamination. The cDNAs were stored at −20°C until use.

### Real Time PCR

Specific primer for TWEAK [32], IL-18 [5], TGF-β1 [24] and Ribosomal protein L13A (RPL13A) [33] were previously published. Primers used for NKp46 (NKp46-s: gactagagagcgagccagca and NKp46-as: aagagtctgtgtgttcagccttc) were designed using the Universal Probe Library Assay Design Center (www.roche-applied-science.com) and sequences were searched against GenBank sequences with the BLAST program to ensure the specificity of primers. Real-time PCR was carried out using a LightCycler 480 apparatus (Roche, Meylan, France). Reactions were set up using the following final concentrations: 0.5 µM of sense and antisens primers, 1X 480 SYBER Green master mix and 4 µl of 1/20 diluted cDNA. Cycling conditions were as follows: denaturation (95°C for 5 min), amplification and quantitation (95°C for 10 s, 60°C for 10 s and 72°C for 15 s) repeated 40 times, a melting curve program (65–95°C with a ramp rate of 2,2°C/s) and a cooling step to 4°C. In addition to the no-reverse transcription and no-template controls, an independently inter-run calibrator (IRC) was included in each RT-PCR assay. This IRC was obtained from Blast-cells for TWEAK, TGF-β and NKp46 and from placenta for IL-18, RPL-13.

In each assay, an aliquot of the IRC cDNA was 20 times diluted and was submitted to the qPCR protocol as the unknown samples. PCR efficiencies for each quantified target and reference were calculated using known serial dilutions of each specific cDNA. Data were analysed using the LightCycler®480 Software release 1.5.0. Each specific target transcription level was normalized to the geometric mean of the transcription levels of the reference gene, using the Advanced Relative Quantification of the LightCycler®480 Software. Each gene specific amplification efficiency was specified. For each sample, results were expressed in concentration ratio (target/reference mRNAs).

### Immunohistochemistry

Micro-explants (on days 1 and 5) were fixed in 4% buffered formaldehyde (96% Phosphate Buffer Saline or PBS) overnight at 4°C. They were washed with PBS, dehydrated in alcohol (70%, 95% and 100% ethanol) before being embedded in paraffin. Paraffin sections (5 µm thick on Superfrost glass slides) were deparaffined in histolemon and re-hydrated with alcohol. Endogenous peroxydases and biotin activities were inhibited by incubation of the slides in a specific solution (Peroxydase Blocking solution, DAKO, Trappes, France and Avidin/Biotin Blocking Vector kit, Abcys, Paris, France) or by incubation with 0.3% hydrogen peroxide. Protein blocking was achieved by a 20 min incubation in PBS (Phosphate Buffer Saline) with 1% of horse serum and 5% human normal serum (blocking serum).

✓ Morphological evaluation: Cytokeratin staining was made using mouse anti-human cytokeratin pan KL1, dilution 1/40 (Clinisciences, Montrouge, France) and vimentin staining by using mouse anti-human vimentin V9, dilution 1/200 (Biogenex, France). KL1and vimentin immunostaining detection was performed using the appropriated secondary antibody from kit Vectastain (Vector ABC, Abcys, Paris, France) revealed with 3,3′ diaminobenzidine (DAB, DAKO, Trappes, France).

✓ NKp46 experiments: Physiological ranges of IL-18 and TWEAK mRNA expression have been previously established in control fertile women [28]. Briefly, TWEAK and IL-18 expression were normalised using geometric mean of three validated references genes (for the endometrium). We then calculated the mean (+/− SEM) of these ratio in our fertile patient group allowing us to obtain the normal range both for IL-18 (3.8 to 6) and TWEAK (4.2–6.5) mRNa expressions. We retrospectively selected endometrial samples with a high IL-18 mRNA expression (over 6.17) and low (below 4.1) or high TWEAK (over 6.86) mRNA expression to compare their NKp46 and TGF-β protein expression. NKp46 staining was made using a polyclonal goat antihuman NKp46 antibody, concentration 4 µg/ml, (R&D Systems, Lille, France). Between each steps the slides were rinsed three times with PBS. NKp46 immunostaining detection was performed using the appropriated secondary antibody from kit Vectatstain (Vector ABC, Abcys, Paris, France) for 20 min followed by incubation with a peroxydase-conjugated streptavidin for 30 minutes and then by use of substrate buffer supplemented with liquid DAB and hydrogen peroxide substrate solution (DAKO, Trappes, France).

Counterstaining was performed with hematoxylin and slides were mounted with Eukitt medium for light microscopy observation. Negative controls were performed both by omission of the primary antibody and by its replacement by an appropriate isotype control. All slides were analysed with a fully automatic virtual slide scanner (DotSlide BX micoscope, Olympus, Hamburg, Germany).

### Statistical analysis

Wilcoxon test was used to compare production of cytokines and growth factors (in different conditions of hormones concentration) and TWEAK, IL-18, NKp46 and TGF-β1 mRNA expression in each culture conditions. The statistical assessments were performed using the StatView software (Abacus Concepts, Inc., CA, USA). Significance was set up at *p*≤0. 05.
